# SimCP-NS: similarity-based copy paste for semi-supervised 3D EM neuron segmentation

**DOI:** 10.1093/bioinformatics/btag560

**Published:** 2026-09-15

**Authors:** Tingting Zhang, Wenjia Meng, Xueshi Yu, Gang Pan, Renmin Han

**Affiliations:** School of Software, Shandong University, Jinan 250101, China; School of Software, Shandong University, Jinan 250101, China; The Interdisciplinary Center, Shandong University, Jinan 250101, China; The College of Computer Science and Technology, Zhejiang University, Hangzhou, Zhejiang 310027, China; The State Key Lab of Brain-Machine Intelligence, Zhejiang University, Hangzhou, Zhejiang 310027, China; College of Medical Information and Engineering, Ningxia Medical University, Yinchuan 750004, China; Research Center for Mathematics and Interdisciplinary Sciences (Ministry of Education Frontiers Science Center for Nonlinear Expectations), Shandong University, Qingdao 266237, China

## Abstract

**Motivation:**

Semi-supervised neuron segmentation in 3D electron microscopy (EM) is important for connectomics because it enables accurate neuron reconstruction while reducing dependence on costly manual annotations. However, existing approaches remain limited by the distribution mismatch between the small labeled dataset and the much larger unlabeled dataset, where the labeled data fail to adequately capture the true data distribution, leading models trained on them to generate low-quality segmentation masks and ultimately degrading overall performance.

**Results:**

To address this issue, we propose a similarity-based copy-paste for semi-supervised 3D EM neuron segmentation (SimCP-NS) method, which employs a similarity-based copy-paste strategy to exchange the least similar labeled and unlabeled sub-volumes, thereby enriching data diversity and mitigating the distribution mismatch. A teacher network is first pre-trained on unlabeled volumes to capture structural priors, which subsequently guides the student segmentation network. During student training, the similarity-based copy-paste mechanism generates hybrid samples and constructs supervision targets by fusing teacher-generated pseudo-labels with ground-truth affinity maps, optimized via a mean squared error loss. Invariant representation learning is further integrated to enhance the robustness of the proposed method. Extensive experiments demonstrate the superior performance of SimCP-NS over existing 3D EM neuron segmentation methods.

**Availability and implementation:**

The source code for SimCP-NS is publicly available at https://github.com/zhangtingting01230123/SimCP-NS.

## 1 Introduction

Neuron segmentation in electron microscopy (EM) volumes is fundamental to connectomics, as it provides the structural basis for reconstructing cell-level neural circuits and understanding the organization of the nervous system (Berning *et al.* 2015, Xu *et al.* 2017, Han *et al.* 2018, Lin *et al.* 2019, Dorkenwald *et al.* 2022, Peddie *et al.* 2022, Sheridan *et al.* 2023). However, neuron segmentation in 3D EM volumes remains challenging due to complex morphologies, ambiguous boundaries, dense cellular distributions, and large fields of view spanning entire volumes. These factors limit the applicability of segmentation methods developed for natural images (Bolya *et al.* 2019, Yi *et al.* 2020), often leading to over-merging or over-segmentation errors when applied to neuron segmentation.

To address this challenge, numerous neuron segmentation approaches have been developed, which largely fall into two categories. Object-tracking-based methods (Januszewski *et al.* 2018, Meirovitch *et al.* 2019) iteratively trace and expand neuron instances from seed points, thereby maintaining strong topological consistency. Boundary-detection-based methods (Lee *et al.* 2017, Zeng *et al.* 2017, Funke *et al.* 2019, Huang *et al.* 2022b, Sheridan *et al.* 2023, Wang *et al.* 2024) predict voxel affinities or boundaries and then assemble them into neuron instances through post-processing (Beier *et al.* 2017, Funke *et al.* 2019). However, these methods depend heavily on high-quality labeled data, yet producing such annotations is extremely difficult and time-consuming due to the dense packing and complex morphology of neurons, which limits the effectiveness of existing approaches (Chen *et al.* 2023, Liu *et al.* 2024).

To reduce the reliance on costly annotations, semi-supervised EM neuron segmentation methods have been developed to leverage both labeled and unlabeled volumes. Specifically, SSNS-Net (Huang *et al*. 2022a) proposes a two-stage semi-supervised framework for neuron segmentation that exploits both labeled and unlabeled data, thereby reducing the dependence on costly manual annotations. Takaya *et al*. (2021) further utilize structural continuity across adjacent EM slices to generate more reliable pseudo-labels, enabling effective use of large unlabeled datasets under limited labeled supervision. However, these approaches still suffer from the distribution mismatch between the small labeled dataset and the much larger unlabeled dataset (Fig. 1a), where the limited labeled data fails to represent the true data distribution, leading segmentation networks trained on them to generate low-quality segmentation masks and thereby undermining overall performance.

**Figure 1:**
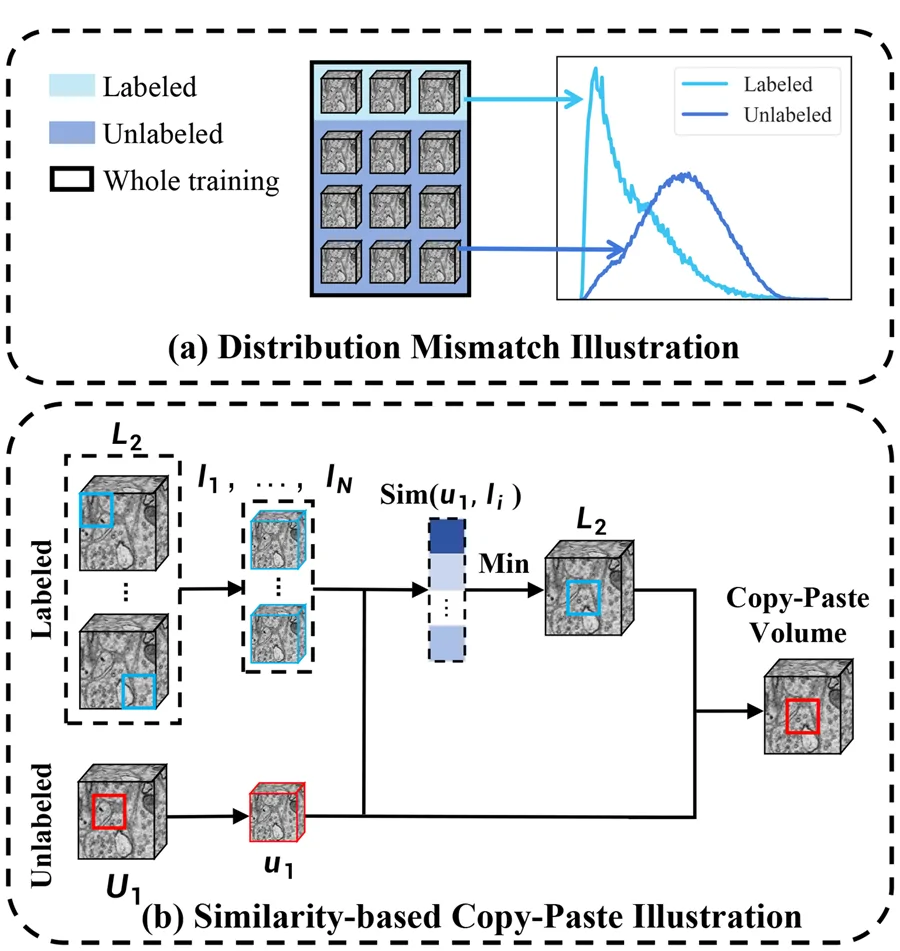
Distribution mismatch and the proposed copy-paste strategy. (a) Labeled–unlabeled mismatch. (b) Brief overview of similarity-based copy-paste. $\text{Sim}(u_{1},l_{i})$ denotes the similarity between sub-volumes $u_{1}$ and $l_{i}$ for i=1,…,N.

In this paper, we propose Similarity-based Copy-Paste for semi-supervised 3D EM Neuron Segmentation (SimCP-NS). SimCP-NS exchanges the least similar labeled and unlabeled sub-volumes to construct hybrid samples, thereby enriching label-informed training data and reducing the distribution mismatch between labeled and unlabeled data. We first pre-train a teacher network on unlabeled volumes to learn structural priors, and then train a student network on both labeled and unlabeled data under the teacher’s guidance. During student training, the copy-paste module identifies the least similar pairs and performs bidirectional exchanges (Fig. 1b), further expanding volume diversity. Supervision for hybrid samples is obtained by fusing teacher-generated pseudo-affinity maps with ground-truth affinities via the same copy-paste operations, enabling effective optimization with a mean squared error loss. In addition, invariant representation learning is incorporated to enhance the robustness and generalization of the student network. Extensive experiments on benchmark datasets demonstrate the effectiveness of the proposed SimCP-NS. Our main contributions are summarized as follows:

- We develop a similarity-based copy-paste augmentation strategy that adaptively selects the least similar sub-volume pairs and performs bidirectional copy-paste between labeled and unlabeled data to generate labeled volumes containing more diverse information.
- We propose SimCP-NS, a semi-supervised neuron segmentation method that combines similarity-based copy-paste augmentation and invariant representation learning to mitigate the distribution mismatch between labeled and unlabeled data and enhance segmentation robustness.
- Extensive evaluations on benchmark datasets show that SimCP-NS outperforms other methods, with ablation studies confirming the effectiveness of its key modules.

## 2 Preliminary

### 2.1 Problem definition

Given an EM volume $V\in R^{D\times H\times W}$, where *D*, *H*, and *W* denote the depth, height, and width of the volume, respectively, the goal of neuron segmentation is to predict a 3D segmentation mask $S\in N^{D\times H\times W}$ that assigns each voxel a unique neuron ID, with background voxels labeled as 0. Existing neuron segmentation methods (Lee *et al.* 2017, Huang *et al.* 2022a,b, Wang *et al.* 2024) typically predict an affinity map *A*, which encodes whether each voxel in the EM volume *V* and its neighboring voxels belong to the same neuron, and which is post-processed (Beier *et al.* 2017, Funke *et al.* 2019) to produce the segmentation mask *S*.

### 2.2 Affinity definition

In neuron segmentation, the segmentation model predicts an affinity map $A\in {\left[0,1\right]}^{3\times D\times H\times W}$, which assigns three affinity values to each voxel in the EM volume *V*, representing the probability that this voxel and its neighboring voxels along the depth, height, and width directions belong to the same neuron. Formally, *A* can be defined as:


(1) $$A(m,i,j,k)=\{\begin{array}{cc} \text{Prob}(V(i,j,k)∼V(i+1,j,k)), & m=0,\\ \text{Prob}(V(i,j,k)∼V(i,j+1,k)), & m=1,\\ \text{Prob}(V(i,j,k)∼V(i,j,k+1)), & m=2, \end{array}$$

where ∼ denotes “the two voxels belong to the same neuron,” and 0≤i<D, 0≤j<H, 0≤k<W.

## 3 Proposed method

This section introduces SimCP-NS, a semi-supervised neuron segmentation method that applies a similarity-based copy-paste strategy to generate hybrid samples that enrich labeled information and mitigate distribution mismatch. We first outline the overall framework and teacher pre-training, then introduce the key components of student training, including similarity-based copy-paste augmentation, invariant representation learning, and the associated loss functions, and finally present the pseudocode for SimCP-NS.

### 3.1 Framework of proposed method

The overall framework of the proposed method is shown in Fig. 2. Labeled and unlabeled volumes are first randomly cropped to obtain sub-volume pairs, with unlabeled pairs passed through the teacher network to generate pseudo-affinity maps. A similarity-based copy-paste operation exchanges the least similar labeled and unlabeled sub-volumes to form hybrid volumes, which are further perturbed and fed into the student network to extract latent representations and predict affinity maps. Supervision targets are then constructed by applying the same copy-paste operation to fuse teacher pseudo-affinities with ground-truth affinities. The student network is optimized with two losses. The first is the MSE loss between predicted affinity maps and supervision targets. The second is the invariant-representation loss that enforces consistency between original and augmented inputs in both latent representations and predicted affinities. The teacher network is updated via the EMA (Tarvainen and Valpola 2017) to ensure stable training.

**Figure 2:**
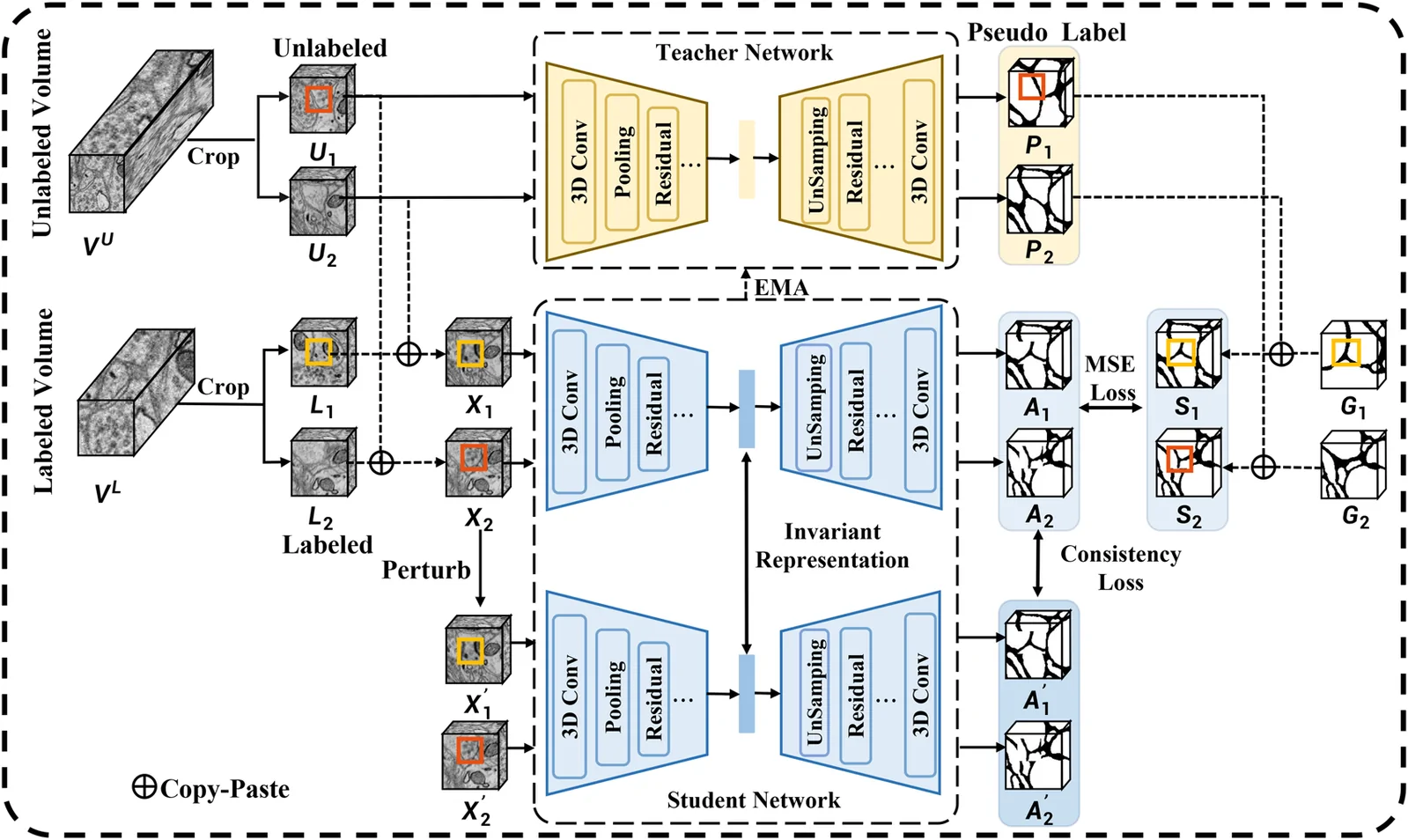
The framework of SimCP-NS. Its detailed descriptions are provided in Section 3.1.

### 3.2 Pre-training of teacher network

Given a small labeled dataset $V_{L}\in R^{D_{L}\times H\times W}$ and a much larger unlabeled dataset $V_{U}\in R^{D_{U}\times H\times W}$, where $D_{L}\ll D_{U}$ due to the high cost of manual annotation, we first pre-train a teacher segmentation network using unlabeled volumes. Specifically, the teacher adopts a 3D ResUNet architecture (Lee *et al.* 2017) with an encoder-decoder structure. The encoder applies 3D convolutions, pooling, and four residual blocks to map an unlabeled sub-volume $U\in R^{d\times h\times w}$ (cropped from $V_{U}$) into a latent representation:


(2) $$z=\text{Enc}(\text{Perturb}(U);\theta_{t}),$$

where Enc(·) denotes the encoder with parameters $\theta_{t}$. Perturb(·) denotes a stochastic perturbation operation for 3D EM sub-volumes, which randomly selects one augmentation from Gaussian blur, Gaussian noise, Cutout, slice misalignment, and contrast variation. The decoder reconstructs the sub-volume from *z*:


(3) $$\hat{U}=\text{Dec}(z;\eta_{t}),$$

where Dec(·) denotes the decoder with parameters $\eta_{t}$. Thus, the full teacher network is:


(4) $$\hat{U}=\text{Seg}(U;\phi_{t})=\text{Dec}(\text{Enc}(\text{Perturb}(U);\theta_{t});\eta_{t}),$$

where $\phi_{t}=\{\theta_{t},\eta_{t}\}$ denotes all network parameters. The teacher segmentation network is optimized by minimizing the reconstruction loss function:


(5) $$L_{\text{rec}}=\sum_{U=\text{Crop}(V_{U})}\|\hat{U}-U{\|}_{2}^{2},$$

where Crop(·) denotes random cropping. With the pre-trained teacher network, the student network $\text{Seg}(\cdot ;\phi_{s})$ is initialized with $\phi_{s}=\{\theta_{s},\eta_{s}\}$, where the encoder weights $\theta_{s}$ are inherited from the teacher encoder $\theta_{t}$.

### 3.3 Similarity-based copy-paste augmentation

In this section, we describe the similarity-based copy-paste augmentation process that integrates complementary structural information from labeled and unlabeled domains to mitigate distribution mismatch, including how dissimilar sub-volume pairs are selected and how bidirectional copy-paste is performed.

#### 3.3.1 Similarity-based copy-paste pair selection

To alleviate the distribution mismatch caused by limited labeled data, we enhance the diversity of labeled-information-containing volumes. The most dissimilar labeled and unlabeled sub-volumes are selected as copy-paste pairs to generate hybrid volumes that integrate complementary features from both domains. Specifically, given a labeled volume $V_{L}\in R^{D_{L}\times H\times W}$ and an unlabeled volume $V_{U}\in R^{D_{U}\times H\times W}$, we randomly crop them to get four volumes:


(6) $$L_{1},L_{2}=\text{Crop}(V_{L}),U_{1},U_{2}=\text{Crop}(V_{U}),$$

where $L_{1}$, $L_{2}$, $U_{1}$, and $U_{2}$ are volumes of identical spatial dimensions $R^{d\times h\times w}$.

**Copy-paste pair selection from** $U_{1}$ **to** $L_{2}$. We first randomly crop a sub-volume $u_{1}\in R^{\beta d\times \beta h\times \beta w}$ from $U_{1}$, where β is a hyperparameter controlling the scale of the cropped region. We then construct a set of *N* candidate sub-volumes $C_{L_{2}}$ by iteratively cropping sub-volumes from $L_{2}$ using a sliding window, which is initialized with its top-left-front corner aligned with that of $L_{2}$ and subsequently moved along the *x*, *y*, and *z* axes with a fixed stride:


(7) $$C_{L_{2}}={\left\{l_{2}^{n}\right\}}_{n=1}^{N},$$

where $l_{2}^{n}$ denotes the *n*-th candidate sub-volume. For each $l_{2}^{n}\in C_{L_{2}}$, we compute its structural similarity with $u_{1}$ and select the one with the lowest similarity as the target sub-volume $l_{2}$ of the labeled volume $L_{2}$ for the copy-paste:


(8) $$l_{2}=\text{arg}\underset{l_{2}^{n}\in C_{L_{2}}}{\min}\text{Sim}(u_{1},l_{2}^{n}),$$

where Sim(·) is the similarity function.

**Copy-paste pair selection from** $L_{1}$ **to** $U_{2}$. Similarly, we first randomly crop a sub-volume $l_{1}\in R^{\beta d\times \beta h\times \beta w}$ from $L_{1}$. In the unlabeled volume $U_{2}$, we initialize a sliding window at the top-left-front corner and apply the same sliding strategy to generate *N* candidate sub-volumes:


(9) $$C_{U_{2}}={\left\{u_{2}^{n}\right\}}_{n=1}^{N}.$$

We then compute the structural similarity between each $u_{2}^{n}\in C_{U_{2}}$ and $l_{1}$, and select the one with the lowest similarity as the target sub-volume $u_{2}$ of the unlabeled volume $U_{2}$:


(10) $$u_{2}=\text{arg}\underset{u_{2}^{n}\in C_{U_{2}}}{\min}\text{Sim}(l_{1},u_{2}^{n}).$$

Finally, we obtain the paired sub-volumes $\left(u_{1},l_{2}\right)$ and $\left(u_{2},l_{1}\right)$, which serve as the copy-paste pairs for the subsequent augmentation operations.

#### 3.3.2 Bidirectional copy-paste and supervision construction

To further improve cross-domain feature integration, we introduce a bidirectional copy-paste strategy, in which the labeled sub-volume is embedded into the unlabeled volume and vice versa. This bidirectional operation increases the diversity of labeled-information-containing volumes and further reduces the distribution mismatch. Given the similarity-based pairs $\left(u_{1},l_{2}\right)$ and $\left(u_{2},l_{1}\right)$ [Equations (8) and (10)], we perform bidirectional copy-paste operations to synthesize two mixed volumes, $X_{1}$ and $X_{2}$.

During the copy-paste process from $U_{1}$ to $L_{2}$, we first construct a modified volume ${\hat{U}}_{1}$ by replacing the voxel values of $U_{1}$ at the spatial location of $l_{2}$ with those from $u_{1}$, and then synthesize the mixed volume $X_{2}$ in a unified form:


(11) $${\hat{U}}_{1}=\text{Replace}(U_{1},\text{pos}(l_{2}),\text{pos}(u_{1})),$$

(12) $$X_{2}=(L_{2}⊙M_{l_{2}})+({\hat{U}}_{1}⊙(1-M_{l_{2}})),$$

where Replace(·) performs voxel substitution, pos(·) denotes the spatial location of a sub-volume, ⊙ is the element-wise multiplication, and “1” denotes an all-one tensor with the same size as the corresponding mask. The mask $M_{l_{2}}\in R^{d\times h\times w}$ has voxel values of 0 within the region of $l_{2}$ and 1 elsewhere. Similarly, the copy-paste process from $L_{1}$ to $U_{2}$ yields:


(13) $${\hat{L}}_{1}=\text{Replace}(L_{1},\text{pos}(u_{2}),\text{pos}(l_{1})),$$

(14) $$X_{1}=(U_{2}⊙M_{u_{2}})+({\hat{L}}_{1}⊙(1-M_{u_{2}})),$$

where $M_{u_{2}}$ is defined analogously. Through these operations, two synthesized volumes $X_{1}$ and $X_{2}$ are obtained.

**Copy-paste volumetric supervision.** Since the constructed volumes include unlabeled volumes ($U_{1}$ and $U_{2}$), the pretrained teacher model $\text{Seg}(\cdot ;\phi_{t})$ is used to generate pseudo-affinity maps $P_{1},P_{2}\in R^{3\times d\times h\times w}$:


(15) $$P_{1}=\text{Seg}(U_{1};\phi_{t}),\;P_{2}=\text{Seg}(U_{2};\phi_{t}).$$

To construct the corresponding supervisory signals, we use the pseudo-label $P_{2}$ and the ground-truth affinity map $G_{1}$ of $L_{1}$ to form $S_{1}$, and the pseudo-label $P_{1}$ with $G_{2}$ to form $S_{2}$:


(16) $$\{\begin{array}{c} {\hat{G}}_{1}=\text{Replace}(G_{1},\text{pos}(u_{2}),\text{pos}(l_{1})),\\ S_{1}=(P_{2}⊙M_{u_{2}})+({\hat{G}}_{1}⊙(1-M_{u_{2}})),\\ {\hat{P}}_{1}=\text{Replace}(P_{1},\text{pos}(l_{2}),\text{pos}(u_{1})),\\ S_{2}=(G_{2}⊙M_{l_{2}})+({\hat{P}}_{1}⊙(1-M_{l_{2}})). \end{array}$$

Here, $G_{1}$ and $G_{2}$ are the ground-truth affinity maps of $L_{1}$ and $L_{2}$, respectively, while $S_{1}$ and $S_{2}$ serve as volumetric supervision for training the student segmentation network.

### 3.4 Invariant representation learning

To improve the robustness of the student network against perturbations, we adopt an invariant representation learning strategy. Specifically, we apply standard data augmentations to the copy-paste volumes $X_{1}$ and $X_{2}$ from Equations (14) and (12), producing their augmented versions $X_{1}^{\prime }$ and $X_{2}^{\prime }$ via perturbation operations:


(17) $$X_{1}^{\prime }=\text{Perturb}(X_{1}),\;X_{2}^{\prime }=\text{Perturb}(X_{2}).$$

The perturbation operation follows Equation (2), where one of the five augmentations is randomly applied to the copy-paste volumes.

Given the copy-paste volumes $X_{1}$ and $X_{2}$ and their augmented versions $X_{1}^{\prime }$ and $X_{2}^{\prime }$, we feed them into the student encoder to obtain the corresponding latent representations:


(18) $$\{\begin{array}{cc} z_{1}=\text{Enc}(X_{1};\theta_{s}), & \;z_{2}=\text{Enc}(X_{2};\theta_{s}),\\ z_{1}^{\prime }=\text{Enc}(X_{1}^{\prime };\theta_{s}), & \;z_{2}^{\prime }=\text{Enc}(X_{2}^{\prime };\theta_{s}). \end{array}$$

To encourage the network to learn invariant representations under data perturbations, we enforce consistency between the representations of the original and augmented pairs $\left(z_{1},z_{1}^{\prime }\right)$ and $\left(z_{2},z_{2}^{\prime }\right)$ as follows:


(19) $$L_{z}=\|z_{1}-z_{1}^{\prime }{\|}_{2}^{2}+\|z_{2}-z_{2}^{\prime }{\|}_{2}^{2},$$

where $\|\cdot {\|}_{2}^{2}$ denotes the squared Euclidean distance. Next, we feed the latent representations $z_{1}$, $z_{2}$, $z_{1}^{\prime }$, and $z_{2}^{\prime }$ into the decoder to generate the corresponding affinity predictions $A_{1}$, $A_{2}$, $A_{1}^{\prime }$, and $A_{2}^{\prime }$:


(20) $$\{\begin{array}{cc} A_{1}=\text{Dec}(z_{1};\eta_{s}), & A_{2}=\text{Dec}(z_{2};\eta_{s}),\\ A_{1}^{\prime }=\text{Dec}(z_{1}^{\prime };\eta_{s}), & A_{2}^{\prime }=\text{Dec}(z_{2}^{\prime };\eta_{s}), \end{array}$$

To further improve robustness, we construct the consistency loss to optimize the student network:


(21) $$L_{\text{cons}}=\frac{1}{N_{u}}\sum_{j=1}^{N_{u}}\left[\|A_{1}^{j}-A_{1}^{\prime j}{\|}_{2}^{2}+\|A_{2}^{j}-A_{2}^{\prime j}{\|}_{2}^{2}\right],$$

where $N_{u}$ denotes the number of training samples, $A_{1}^{j}$, $A_{2}^{j}$, $A_{1}^{\prime j}$, $A_{2}^{\prime j}$ denote the affinity predictions of the *j*th sample before and after augmentation. Finally, combining the latent representation loss $L_{z}$ in Equation (19) and the consistency loss $L_{\text{cons}}$ in Equation (21), the loss for learning invariant representations is formulated as:


(22) $$L_{\text{inv}}=\kappa L_{\text{cons}}+\nu L_{z},$$

where κ and ν are hyperparameters.

### 3.5 Loss functions

To prevent unreliable pseudo-labels from dominating the optimization, we first define a basic mean squared error (MSE) loss L(A,S) to measure the voxel-wise difference between a predicted affinity map *A* and its target *S*:


(23) $$L(A,S)=\frac{1}{N_{u}}\sum_{j=1}^{N_{u}}\left[\|A_{1}^{j}-S_{1}^{j}{\|}_{2}^{2}+\|A_{2}^{j}-S_{2}^{j}{\|}_{2}^{2}\right],$$

where $A_{1}^{j}$ and $A_{2}^{j}$ are the predicted affinity maps for the *j*-th training sample, and $S_{1}^{j}$ and $S_{2}^{j}$ are the corresponding supervisiory affinity targets. Based on the MSE loss in Equation (23) and invariant representation loss in Equation (22), the total loss for the student network is:


(24) $$L_{\text{total}}=L_{\text{inv}}+\lambda L(A,S)$$

where λ is a hyperparameter. Additionally, during student training, the teacher network is updated using the EMA (Tarvainen and Valpola 2017) for stable synchronization.

### 3.6 Pseudocode

The overall procedure of SimCP-NS is summarized in Algorithm 1, with details in the Supplementary material, available as supplementary data at *Bioinformatics* online.

Algorithm 1 Proposed SimCP-NS

**Input**: Labeled dataset $V_{L}$ with ground-truth $A^{GT}$; unlabeled dataset $V_{U}$; test dataset $V_{T}$

**Output**: Neuron segmentation *S*

1: **I. Teacher Network Pre-training** 

2: Initialize teacher network $\text{Seg}(\cdot ;\phi_{t})$ with $\phi_{t}=\{\theta_{t},\eta_{t}\}$

3: **while** not done **do** 

4:  Crop unlabeled volume *U* from $V_{U}$

5:  Reconstruct $\hat{U}$ using Equation (4)

6:  Optimize $\phi_{t}$ using $L_{\text{rec}}$ in Equation (5)

7: **end while** 

8: **II. Student Network Training** 

9: Initialize student network $\text{Seg}(\cdot ;\phi_{s})$ with $\phi_{s}=\{\theta_{s},\eta_{s}\}$ and $\theta_{s}=\theta_{t}$

10: **while** not done **do** 

11: Obtain copy-paste volumes $X_{1}$ and $X_{2}$ using Equations (14) and (12)

12: Generate pseudo-labels $P_{1}$ and $P_{2}$ using Equation (15)

13:  Construct supervisions $S_{1}$ and $S_{2}$ using Equation (16)

14:  Optimize $\phi_{s}$ using $L_{\text{total}}$ in Equation (24)

15:  Update $\phi_{t}$ by EMA according to $\theta_{s}$

16: **end while** 

17: **III. Testing** 

18: Crop test volume *T* from $V_{T}$

19: Compute affinity map $A\leftarrow \text{Seg}(T;\phi_{s})$

20: Post-process *A* to obtain final segmentation *S*

## 4 Experiments

In this section, we conduct experiments to evaluate the proposed SimCP-NS. We first introduce our experimental setup, including the datasets, metrics, and implementation details. Then, we compare our proposed method with other state-of-the-art methods. We also visualize the segmentation results to further validate SimCP-NS. Finally, we perform ablation studies to verify key components.

### 4.1 Experimental setup

**Datasets.** Experiments are conducted on two standard EM neuron segmentation benchmarks: AC3/AC4 (Kasthuri *et al.* 2015) and CREMI (Funke *et al.* 2016). AC3 and AC4 originate from the mouse somatosensory cortex (Kasthuri *et al.* 2015), containing 256 and 100 consecutive 1024×1024 slices, respectively. Following common practice, the top five AC3 slices serve as labeled data, the bottom 50 slices of AC3 and AC4 are used for testing, and 200 slices from AC3/AC4 are used as unlabeled data for semi-supervised learning. CREMI consists of 3D electron microscopy volumes of adult Drosophila brain tissue at $4\times 4\times 40{\text{nm}}^{3}$. Due to space limitations, we present results on the representative CREMI-C dataset in the main paper. For CREMI-C, we use the first five labeled slices for training, the bottom 50 for testing, and 100 unlabeled slices for semi-supervised learning.

**Metrics and implementation details.** Neuron segmentation is evaluated using Variation of Information (VOI) (Nunez-Iglesias *et al.* 2013) and Adapted Rand Error (ARAND) (Arganda-Carreras *et al.* 2015). VOI measures dissimilarity between segmentation masks and can be split into ${\text{VOI}}_{\text{split}}$ and ${\text{VOI}}_{\text{merge}}$ to assess the extent of over-splitting and over-merging error. ARAND accounts for uneven object sizes and quantifies agreement with ground truth (Rand 1971). Lower values indicate better accuracy. During training, the labeled sub-volumes $L_{1}$ and $L_{2}$, and the unlabeled sub-volumes $U_{1}$ and $U_{2}$, are randomly cropped to the same spatial size of 18×160×160. During inference, predicted affinity maps are converted into neuron segmentation masks using the Waterz (Funke *et al.* 2019) and LMC (Beier *et al.* 2017) post-processing algorithms. More implementation details and our code are provided in the Supplementary material, available as supplementary data at *Bioinformatics* online.

### 4.2 Comparison with state-of-the-art methods

To evaluate the effectiveness of the proposed SimCP-NS, we compare it with state-of-the-art (SOTA) methods on three benchmark datasets, AC3, AC4, and CREMI-C, as shown in Table 1. SimCP-NS consistently achieves strong performance across most settings. On the AC3 dataset, SimCP-NS combined with Waterz attains the best overall results, achieving a VOI of 1.048 and an ARAND of 0.099. Compared with the second-best method, SSNS-Net, our approach reduces the VOI by 0.181 (14.7%) and ARAND by 0.021 (17.5%). On the AC4 dataset, SimCP-NS also demonstrates clear advantages, obtaining a 1.223/0.267 (VOI/ARAND) with Waterz and 1.209/0.126 with LMC, achieving the best performance under both post-processing pipelines. On the CREMI-C dataset, SimCP-NS combined with both post-processing pipelines also achieves competitive results. With Waterz, it obtains a VOI/ARAND of 1.611/0.159, while with LMC, it achieves 1.592/0.152. These consistent results across two post-processing strategies indicate that SimCP-NS performs reliably on the CREMI-C dataset. Overall, SimCP-NS demonstrates superior performance across different datasets and post-processing configurations.

**Table 1:** Segmentation results of different methods on AC3, AC4, and CREMI-C using Waterz and LMC.^a^

Post.	**Method**		**AC3**				**AC4**				**CREMI-C**			
			**VOI** ${}_{\text{split}}↓$	**VOI** ${}_{\text{merge}}↓$	**VOI** ↓	**ARAND** ↓	**VOI** ${}_{\text{split}}↓$	**VOI** ${}_{\text{merge}}↓$	**VOI** ↓	**ARAND** ↓	**VOI** ${}_{\text{split}}↓$	**VOI** ${}_{\text{merge}}↓$	**VOI** ↓	**ARAND** ↓
Waterz	*Sup.*	Superhuman (Lee *et al.* 2017)	0.232	5.698	5.930	0.917	0.285	5.983	6.268	0.946	1.175	1.448	2.623	0.467
		PEA (Huang *et al.* 2022b)	0.658	3.203	3.861	0.820	0.542	4.427	4.969	0.921	1.201	2.298	3.499	0.603
		CAD (Liu *et al.* 2024)	0.702	4.607	5.309	0.887	0.438	5.645	6.083	0.942	0.957	3.051	4.008	0.729
	*Semi.*	UA-MT (Yu *et al.* 2019)	0.720	1.860	2.580	0.426	0.794	2.240	3.034	0.662	0.978	0.863	1.841	0.227
		SASSNet (Li *et al.* 2020)	1.160	1.180	2.340	0.324	1.314	1.368	2.682	0.518	0.935	0.814	1.749	0.193
		SSNS-Net (Huang *et al.* 2022a)	0.702	0.527	1.229	0.120	0.578	0.765	1.343	0.325	0.934	0.775	1.709	0.175
		MCF (Wang *et al.* 2023)	0.724	2.365	3.089	0.678	0.583	2.441	3.024	0.754	0.938	0.916	1.854	0.215
		ABD (Chi *et al.* 2024)	1.565	0.694	2.259	0.206	0.813	2.330	3.143	0.627	0.904	0.862	1.766	0.223
		DCMamba (Li *et al.* 2026)	0.648	1.181	1.829	0.386	0.536	1.487	2.023	0.553	0.933	0.866	1.799	0.185
		Ours	0.570	0.478	**1.048**	**0.099**	0.767	0.456	**1.223**	**0.267**	0.909	0.702	**1.611**	**0.159**
LMC	*Sup.*	Superhuman (Lee *et al.* 2017)	0.601	4.788	5.389	0.873	0.456	5.502	5.958	0.940	1.202	0.668	1.870	0.193
		PEA (Huang *et al.* 2022b)	0.729	2.404	3.133	0.677	0.628	3.949	4.577	0.905	1.337	1.791	3.128	0.470
		CAD (Liu *et al.* 2024)	1.140	1.186	2.326	0.248	1.263	2.785	4.048	0.801	1.200	1.032	2.232	0.306
	*Semi.*	UA-MT (Yu *et al.* 2019)	1.179	1.167	2.346	0.349	1.167	1.428	2.595	0.573	1.438	0.410	1.848	0.268
		SASSNet (Li *et al.* 2020)	2.023	0.508	2.531	0.291	2.409	0.612	3.021	0.472	1.360	0.417	1.777	0.233
		SSNS-Net (Huang *et al.* 2022a)	0.969	0.295	1.264	0.103	0.952	0.335	1.287	0.136	1.208	0.378	**1.586**	0.157
		MCF (Wang *et al.* 2023)	0.923	1.055	1.978	0.327	0.845	1.656	2.501	0.675	1.312	0.802	2.114	0.254
		ABD (Chi *et al.* 2024)	0.969	0.569	1.538	0.183	1.167	1.428	2.595	0.573	1.267	0.673	1.940	0.214
		DCMamba (Li *et al.* 2026)	0.987	0.373	1.360	0.117	0.887	0.679	1.566	0.423	1.254	0.647	1.901	0.236
		Ours	0.861	0.357	**1.218**	**0.098**	0.882	0.327	**1.209**	**0.126**	1.224	0.368	1.592	**0.152**

a Best and second-best are highlighted in **bold** and underlined. *Sup.*/*Semi.* denotes supervised/semi-supervised methods. Results for UA-MT, SASSNet, and SSNS-Net are from Huang *et al.* (2022a); others are reproduced using the authors’ official codes.

### 4.3 Visualization of segmentation results

To further evaluate the segmentation performance of our method, we compare its visual results with those of other neuron segmentation methods from both 2D and 3D perspectives in Figs 3 and 4 on the AC3, AC 4, and CREMI-C datasets. Figure 3 presents the 2D affinity map predictions and instance segmentation visualizations of SimCP-NS and other competing methods. For each dataset, the first row shows the affinity maps, and the second row shows the corresponding instance segmentation results. Compared with other methods, our proposed SimCP-NS produces higher-quality affinity predictions and 2D segmentations, effectively reducing severe over-splitting (yellow boxes) and over-merging (red boxes) errors. Figure 4 presents the 3D segmentation results of different methods on the three datasets. These results demonstrate that our method reconstructs more accurate 3D neuronal structures, with significantly fewer over-splitting (yellow arrows) and over-merging (red arrows) errors than those of competing methods. These visualizations from both 2D and 3D perspectives further validate the effectiveness of our method, demonstrating its ability to achieve more accurate neuron segmentation.

**Figure 3:**
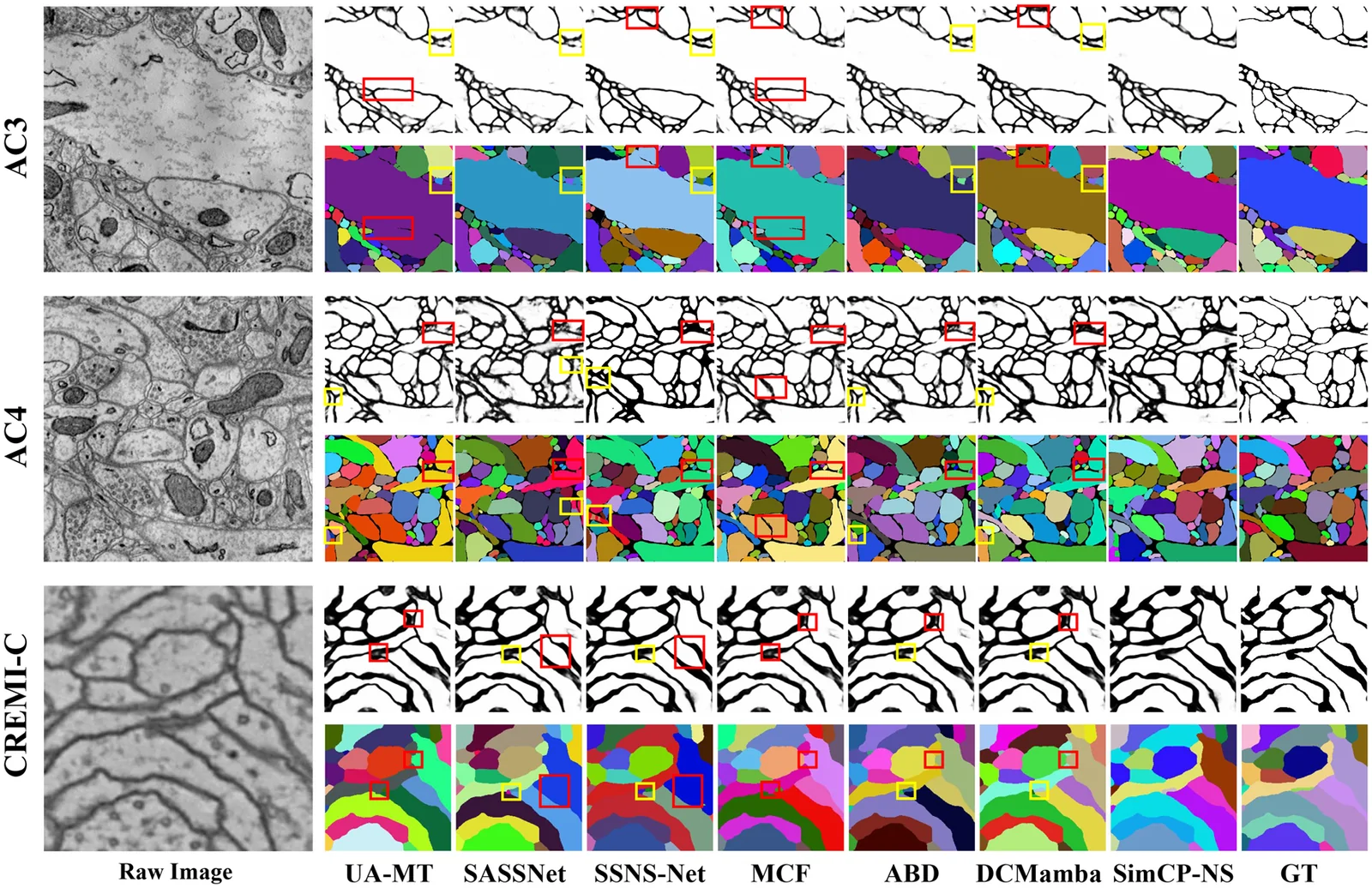
2D visual comparisons of segmentation results on the AC3, AC4, and CREMI-C datasets, including the predicted affinity maps and corresponding neuron segmentations. Red and yellow boxes highlight over-merging and over-splitting errors. The visualizations were generated using the released code from the original papers.

**Figure 4:**
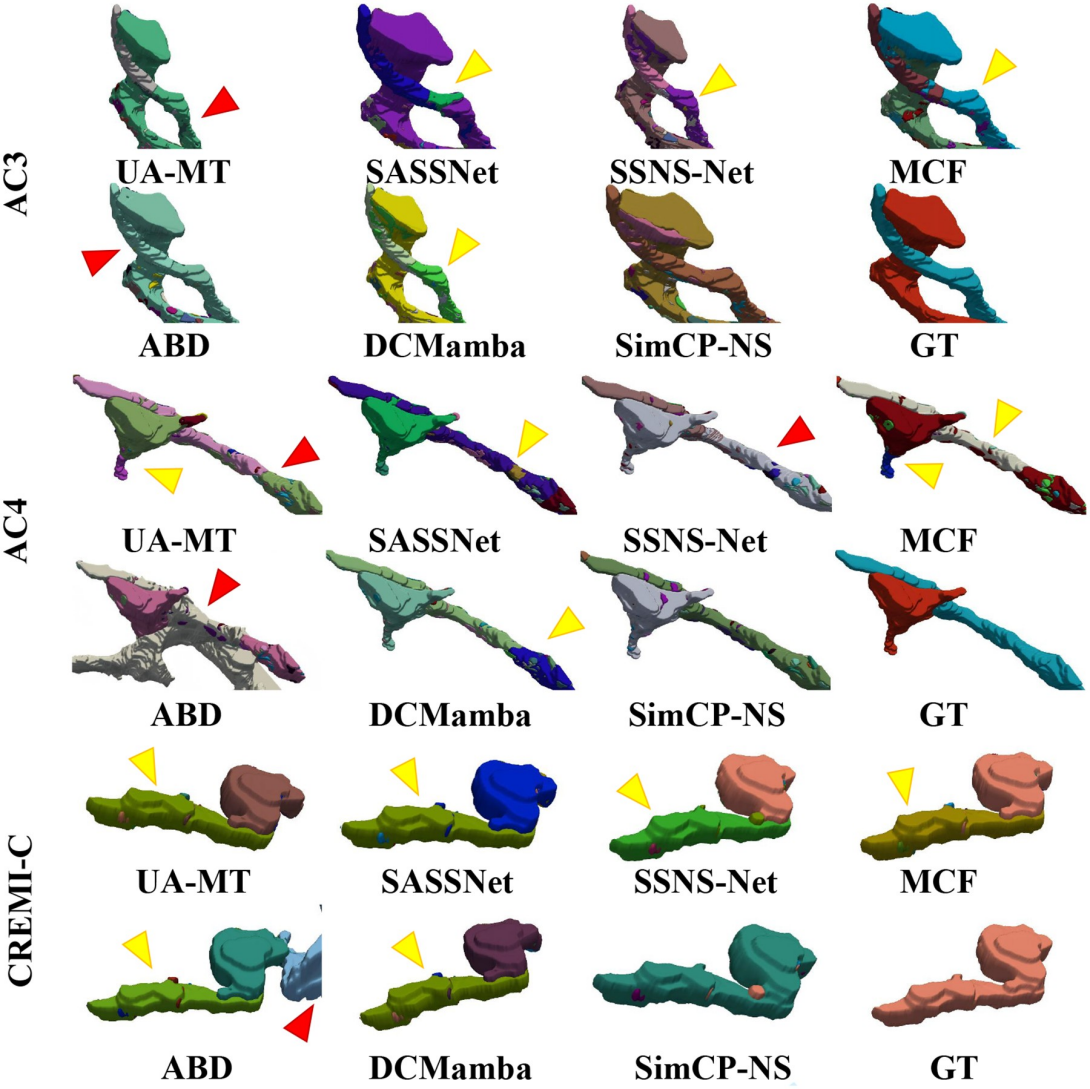
3D visual comparisons of segmentation results on the AC3, AC4, and CREMI-C datasets. Different colors represent different neuron instances, while yellow and red arrows indicate split and merge errors, respectively. The visualizations were generated using the released code from the original papers.

### 4.4 Ablation study

In this section, we present ablation studies on the key components of our method, including invariant representation loss ($L_{\text{inv}}$), bidirectional copy-paste (BCP), and the similarity metric used for copy-paste pair selection. Additional ablation results are provided in the Supplementary material, available as supplementary data at *Bioinformatics* online for completeness.

**Effect of invariant representation loss.** To validate the effectiveness of the invariant representation loss ($L_{\text{inv}}$), we compare the segmentation performance of our method with and without $L_{\text{inv}}$ (w/o $L_{\text{inv}}$) on the AC3 dataset, as shown in Table 2. With $L_{\text{inv}}$, our method achieves a VOI of 1.048 and an ARAND of 0.099, representing improvements of 0.153 and 0.042, respectively, over the variant without $L_{\text{inv}}$. As shown in the first column of Fig. 5, the method without $L_{\text{inv}}$ exhibits noticeable overfitting and over-segmentation compared with our method with $L_{\text{inv}}$. These results demonstrate the effectiveness of the invariant representation loss in improving segmentation performance.

**Figure 5:**
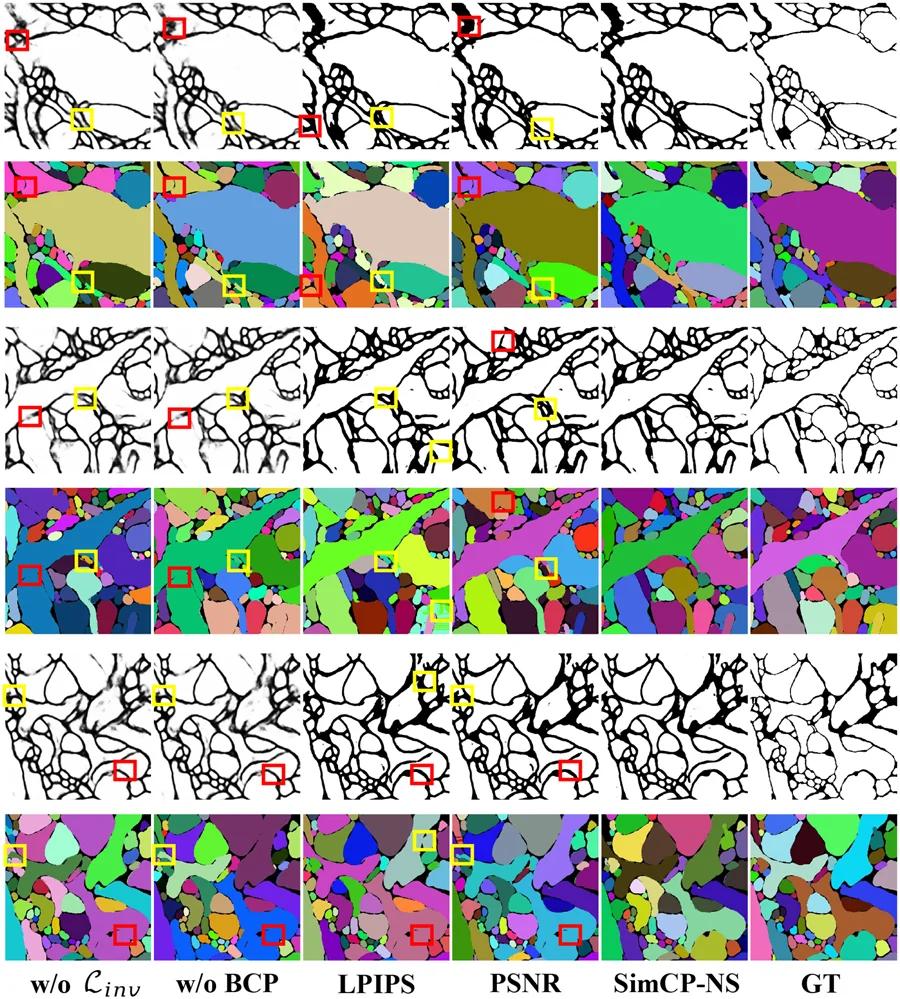
2D visual comparisons of the ablation studies on the AC3 dataset with Waterz post-processing.

**Table 2:** The results of the ablation study under different settings (w/o $L_{\text{inv}}$ and BCP) in our method on the AC3 dataset.

Method	BCP	$L_{\text{inv}}$	**Waterz**		**LMC**	
			**VOI** ↓	**ARAND** ↓	**VOI** ↓	**ARAND** ↓
w/o $L_{\text{inv}}$	✓	×	1.201	0.141	1.425	0.122
w/o BCP	×	✓	1.689	0.231	1.594	0.125
w/BCP + $L_{\text{inv}}$	✓	✓	**1.048**	**0.099**	**1.218**	**0.098**

The best results are highlighted in bold.

**Effect of bidirectional copy-paste.** To validate the effectiveness of the bidirectional copy-paste (BCP) strategy, we compare the segmentation performance of our method with and without the BCP strategy (w/o BCP) on the AC3 dataset, as shown in Table 2. With the BCP strategy, our method achieves a VOI of 1.048 and an ARAND of 0.099, improving by 0.641 and 0.132, respectively, compared with the variant without BCP. Without BCP (the second column of Fig. 5), the method suffers from overfitting and over-segmentation. Overall, the results in Table 2 and Fig. 5 demonstrate the effectiveness of the bidirectional copy-paste strategy.

**Effect of metric.** To validate the effectiveness of using SSIM (Wang *et al.* 2004) as the similarity metric in our copy-paste method, we compare the segmentation performance achieved with different representative similarity metrics. As shown in Table 3, our method using SSIM as the similarity metric to select copy-paste pairs, achieves a VOI of 1.048 and an ARAND of 0.099, outperforming other similarity metrics: Learned Perceptual Image Patch Similarity (LPIPS) (Zhang *et al.* 2018), and Peak Signal-to-Noise Ratio (PSNR). Moreover, as shown in Fig. 5, the SSIM-based SimCP-NS yields more precise segmentation results than the LPIPS- and PSNR-based variants. The results in Table 3 and Fig. 5 validate the effectiveness of adopting SSIM as the similarity metric.

**Table 3:** Ablation study of different similarity metrics for copy-paste pair selection on the AC3 dataset.

Metric	**Waterz**		**LMC**	
	**VOI** ↓	**ARAND** ↓	**VOI** ↓	**ARAND** ↓
LPIPS	1.162	0.136	1.238	0.120
PSNR	1.185	0.130	1.262	0.132
SSIM	**1.048**	**0.099**	**1.218**	**0.098**

The best results are highlighted in bold.

## 5 Conclusion

In this paper, we propose SimCP-NS, a semi-supervised 3D EM neuron segmentation method that employs a similarity-based copy-paste strategy to generate hybrid volumes by exchanging minimally similar sub-volumes, effectively reducing the distribution gap between labeled and unlabeled data. The teacher network, pretrained on unlabeled volumes, provides pseudo-affinity supervision for the student. During training, the student leverages the copy-paste mechanism to form hybrid volumes. Data augmentation and invariant representation learning further enhance robustness. Extensive evaluations on benchmark datasets demonstrate that SimCP-NS achieves superior segmentation accuracy and reliable generalization compared with existing neuron segmentation methods, highlighting its effectiveness for semi-supervised 3D neuron segmentation.

## Supplementary Material

btag560_Supplementary_Data

## Data Availability

The datasets underlying this article are publicly available. The AC3/AC4 dataset is available at https://software.rc.fas.harvard.edu/lichtman/vast/AC3AC4Package.zip, and the CREMI dataset is available at https://cremi.org/data/. No new datasets were generated as part of this study.
