# Supplementary material for: SimCP-NS: similarity-based copy paste for semi-supervised 3D EM neuron segmentation
Source: Bioinformatics. 2026 Sep 15;42(9):btag560. doi: 10.1093/bioinformatics/btag560 (PMC13631923; doi:10.1093/bioinformatics/btag560)
Supplement: Supplementary file 1 [file btag560_Supplementary_Data.pdf]

Supplementary Material for SimCP-NS: Similarity-based Copy Paste for Semi-Supervised 3D EM Neuron Segmentation

The supplementary material provides expanded experimental results, additional visual comparisons, extended ablation studies, and detailed implementation information, whereas the main manuscript presents the principal findings and core methodological descriptions due to page constraints. Specifically, we first present additional quantitative comparisons with state-of-the-art methods on the *CREMI-A* and *CREMI-B* datasets, with extensive 2D and 3D visualizations. *To more comprehensively demonstrate the robustness of our approach, we further include visual comparisons with supervised learning-based neuron segmentation methods (Superhuman, PEA, and CAD), as the qualitative results in the main paper are limited to semi-supervised methods (UA-MT, SASSNet, SSNS-Net, MCF, ABD and DCMamba) due to space limitations.* We then introduce two key ablation studies: one examining the contributions of the bidirectional copy-paste mechanism and the similarity-based sub-volume pair selection, and the other analyzing the effect of different crop-size scales on segmentation performance. Finally, we provide comprehensive implementation details, including network architecture, training hyperparameters, data augmentation strategies, inference configurations, and an expanded explanation of the SimCP-NS pseudocode, clarifying the execution flow and interactions among the major components of the framework.

Comparison with State-of-the-art Methods on CREMI-A and CREMI-B

Quantitative Segmentation Results. To further validate the effectiveness of SimCP-NS, we compare it with representative SOTA methods on the CREMI-A and CREMI-B datasets under both Waterz and LMC post-processing, as summarized in Table S1. On the CREMI-A dataset, SimCP-NS combined with Waterz achieves the best overall performance, obtaining a VOI of 1.057 and an ARAND of 0.249. Compared with the second-best method, SSNS-Net, SimCP-NS reduces VOI by 0.198 (from 1.255 to 1.057, a 15.8% relative improvement) and ARAND by 0.140 (from 0.389 to 0.249, a 36.0% relative improvement). Under the LMC, our method attains the lowest ARAND of 0.191 while achieving the second-best VOI of 1.199, indicating that SimCP-NS remains highly competitive when switching the post-processing strategy. On the CREMI-B dataset, SimCP-NS also demonstrates clear advantages. With Waterz, it achieves a VOI/ARAND of 1.683/0.152. This corresponds to a VOI reduction of 0.335 (16.6%) and an ARAND reduction of 0.158 (51.0%) compared to the second-best method. With LMC, SimCP-NS achieves the best VOI (1.963) and the second-best ARAND (0.146), improving VOI by 0.182 (8.5%) compared to SSNS-Net. These results on CREMI-A and CREMI-B, under two distinct post-processing pipelines, demonstrate that SimCP-NS provides robust and competitive performance across different datasets and evaluation settings.

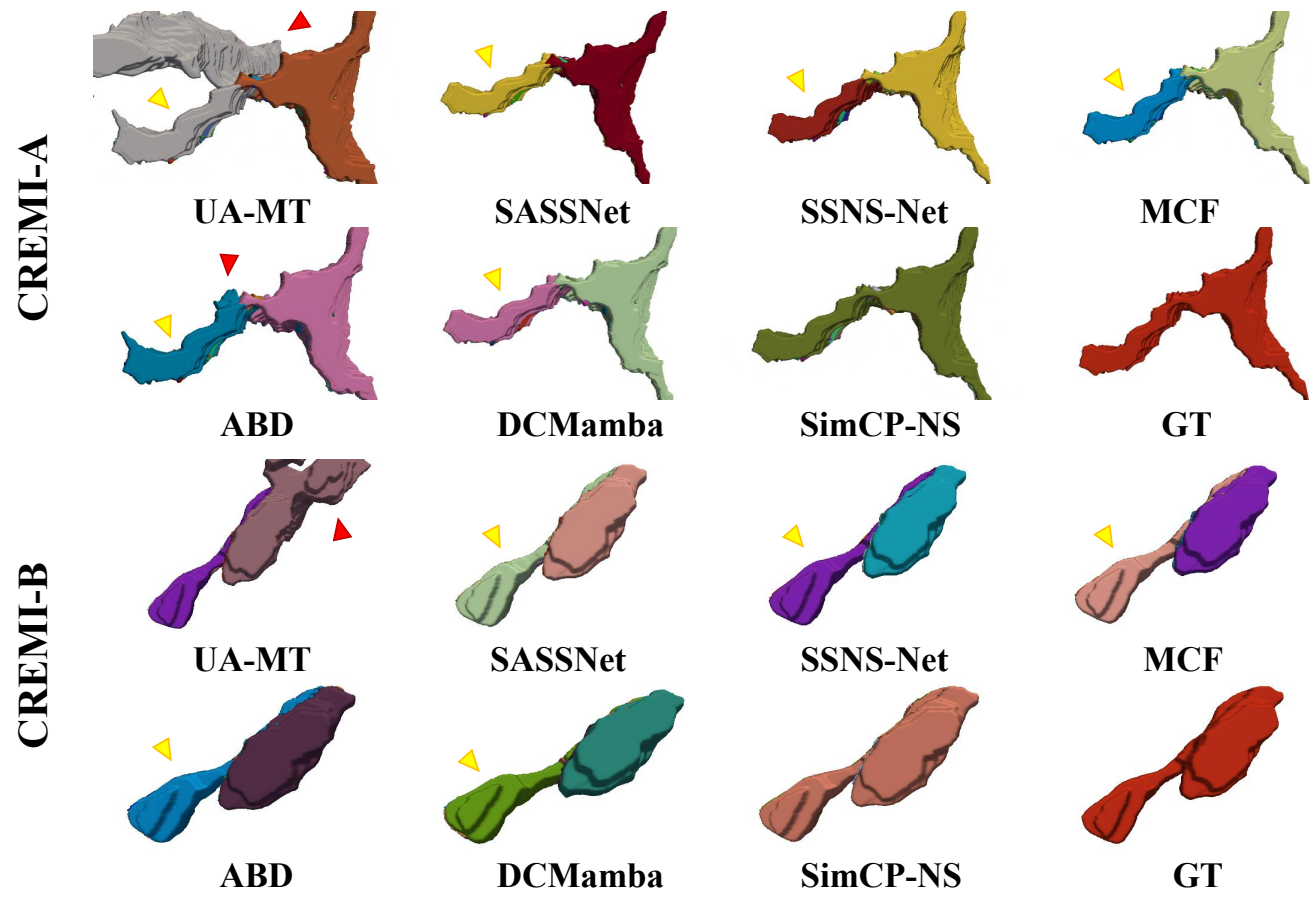

Figure S1 3D visual comparisons of segmentation results on the CREMI-A and CREMI-B datasets. In each sub-figure, different colors represent different neuron instances, while yellow and red arrows indicate split and merge errors in the 3D structures, respectively.

Visualization of Segmentation Results. To further assess the segmentation performance of our semi-supervised approach, we provide qualitative comparisons with other representative semi-supervised neuron segmentation methods (*UA-MT, SASSNet, SSNS-Net, MCF, ABD and DCMamba*) on the CREMI-A and CREMI-B datasets, as shown in Figure S2 and Figure S1. Specifically, Figure S2 presents the 2D affinity map predictions and instance segmentation visualizations of SimCP-NS and competing methods on the CREMI-A and CREMI-B datasets. For each dataset, the first row shows the predicted affinity maps, and the second row shows the corresponding 2D instance segmentation results. Compared with other methods, SimCP-NS yields cleaner affinity maps and more accurate 2D segmentations, substantially alleviating severe over-splitting (highlighted by yellow boxes) and over-merging (highlighted by red boxes) errors. Figure S1 shows the 3D segmentation results of different methods on the CREMI-A and CREMI-B volumes. From the 3D perspective, our method reconstructs more continuous and faithful neuronal structures, with noticeably fewer over-splitting (yellow arrows) and over-merging (red arrows) errors than the competing approaches. These 2D and 3D visualizations further validate the effectiveness and robustness of SimCP-NS on the CREMI-A and CREMI-B datasets, demonstrating its ability to produce more accurate neuron segmentation.

Visual Comparison with Supervised Neuron Segmentation Methods

Owing to space limitations, the qualitative results presented in the main paper focus on semi-supervised neuron segmentation methods (ABD, DCMamba, MCF, UA-MT, SASSNet, and SSNS-Net). To more comprehensively validate the effectiveness of our approach, this section further provides visual comparisons with representative

Table S1. Comparison of segmentation performance of different methods on the CREMI-A and CREMI-B datasets using Waterz and LMC post-processing. The best and second-best results are highlighted in **bold** and underlined, respectively. Note that "*Sup.*" refers to supervised learning-based neuron segmentation methods, while "*Semi.*" refers to semi-supervised learning-based neuron segmentation methods.

Post.		Method	CREMI-A				CREMI-B			
			$VOI_{split}\downarrow$	$VOI_{merge}\downarrow$	VOI↓	ARAND↓	$VOI_{split}\downarrow$	$VOI_{merge}\downarrow$	VOI↓	ARAND↓
Waterz	*Sup.*	Superhuman Lee et al. (2017)	0.562	0.719	1.281	0.455	1.268	1.063	2.331	0.320
		PEA Huang et al. (2022b)	0.645	1.773	2.418	0.819	0.969	1.251	2.220	0.372
		CAD Liu et al. (2024)	0.705	2.120	2.825	0.839	1.224	2.638	3.862	0.681
	Semi.	UA-MT Yu et al. (2019)	0.734	1.307	2.041	0.687	0.726	2.008	2.734	0.513
		SASSNet Li et al. (2020)	0.938	0.839	1.777	0.674	0.699	2.013	2.712	0.561
		SSNS-Net Huang et al. (2022b)	0.433	0.822	1.255	0.389	0.612	1.406	2.018	0.310
		MCF Wang et al. (2023)	0.498	1.145	1.643	0.628	0.696	1.967	2.663	0.436
		ABD Chi et al. (2024)	0.865	0.818	1.683	0.602	0.703	1.890	2.593	0.474
		DCMamba Li et al. (2025)	0.454	1.066	1.520	0.656	0.736	1.778	2.514	0.442
		Ours	0.525	0.532	**1.057**	**0.249**	0.967	0.716	**1.683**	**0.152**
LMC	*Sup.*	SuperhumanLee et al. (2017)	0.781	0.550	1.331	0.282	1.433	0.785	2.218	0.239
		PEA Huang et al. (2022b)	0.695	1.159	1.854	0.674	1.234	0.917	2.151	0.251
		CAD Liu et al. (2024)	0.593	0.666	1.259	0.306	1.449	0.939	2.388	0.276
	Semi.	UA-MT Yu et al. (2019)	0.989	0.840	1.829	0.701	0.893	1.983	2.876	0.497
		SASSNet Li et al. (2020)	0.953	0.724	1.677	0.576	0.672	1.994	2.666	0.531
		SSNS-Net Huang et al. (2022a)	0.603	0.454	**1.057**	0.215	1.236	0.909	2.145	**0.132**
		MCF Wang et al. (2023)	0.782	0.861	1.643	0.421	0.683	1.846	2.529	0.437
		ABD Chi et al. (2024)	0.673	0.857	1.530	0.387	0.721	1.893	2.614	0.324
		DCMamba Li et al. (2025)	0.679	0.766	1.445	0.257	0.722	1.727	2.449	0.245
		Ours	0.827	0.372	1.199	**0.191**	1.579	0.384	**1.963**	0.146

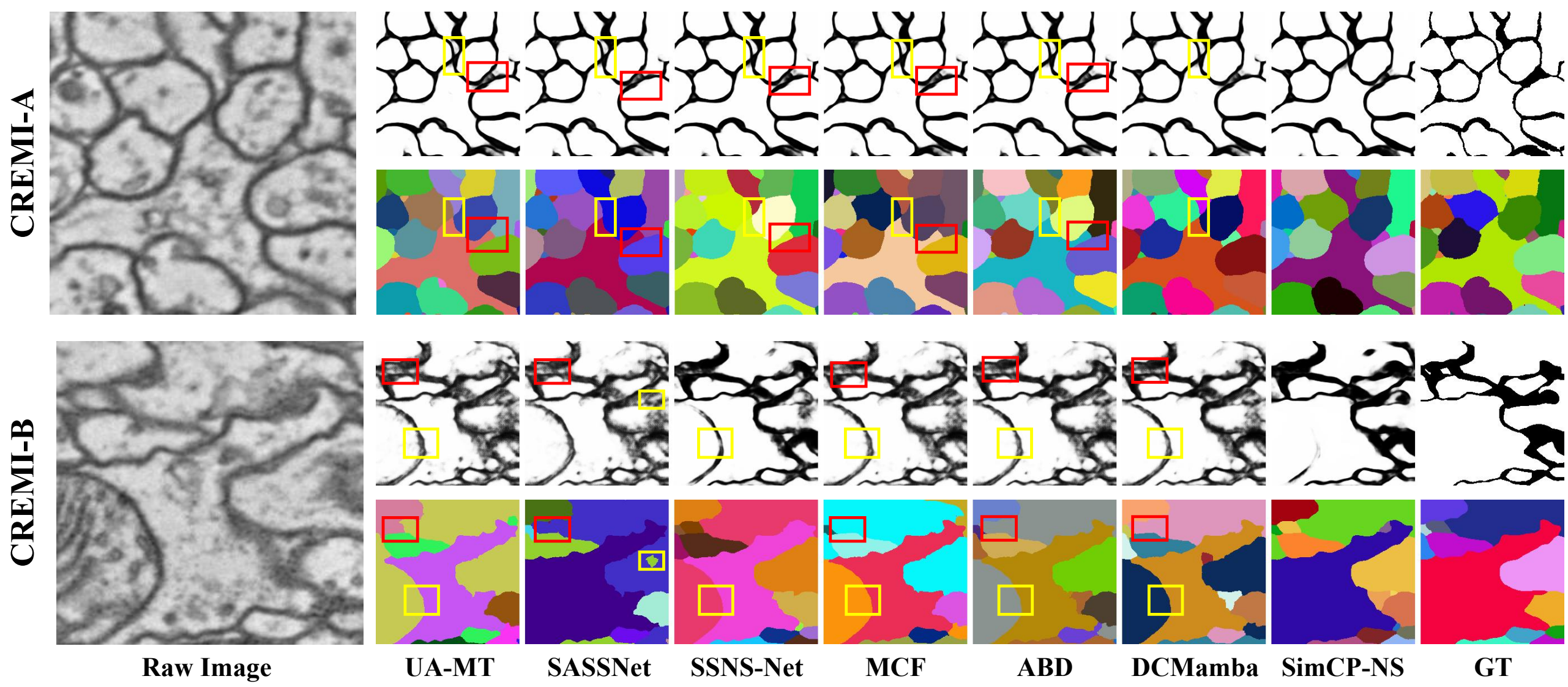

Figure S2 2D visual comparisons of segmentation results on the CREMI-A and CREMI-B datasets. The first row shows the predicted affinity maps, and the second row shows the corresponding neuron segmentations. Red and yellow boxes highlight over-merging and over-splitting errors, respectively.

supervised methods, including Superhuman, PEA, and CAD. The 2D qualitative results on the AC3, AC4, and CREMI datasets are shown in Figure S4, where red and yellow boxes denote over-merging and over-splitting errors, respectively. While supervised methods generally achieve strong performance, our approach substantially reduces these errors and produces more accurate and coherent segmentation boundaries. Figure S3 presents the corresponding 3D segmentation results, which more clearly reveal the continuity and global morphology of neuronal processes. As indicated by the yellow and red arrows, our method better preserves structural continuity and mitigates fragmentation and leakage. These comparisons demonstrate the robustness of our approach in reducing common segmentation failures such as over-merging and over-splitting and highlight

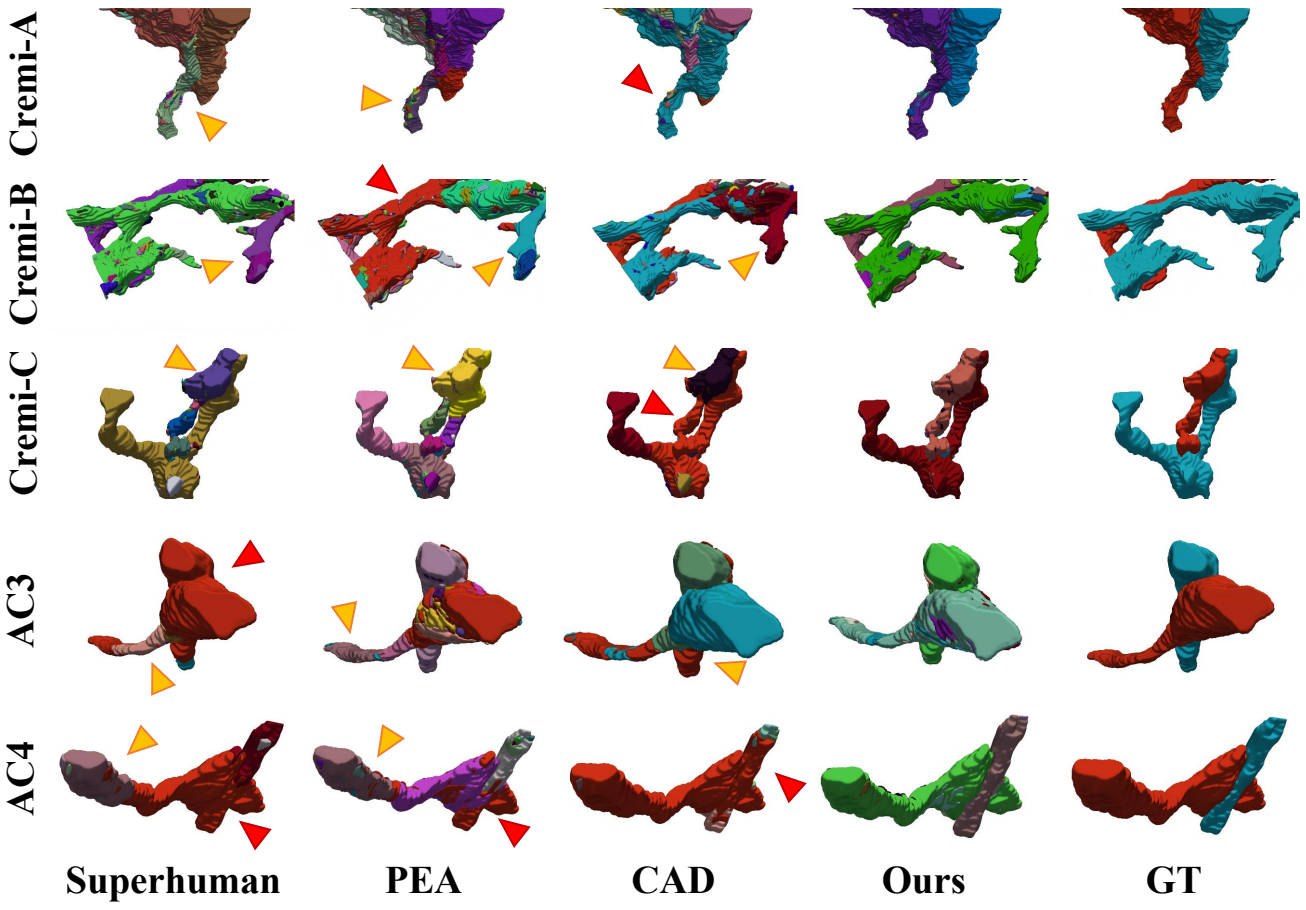

Figure S3 3D visual comparisons with supervised learning-based neuron segmentation methods on the CREMI and AC3/4 datasets. Yellow and red arrows indicate over-splitting and over-merging errors, respectively.

its ability to deliver more precise and topologically consistent neuron segmentation.

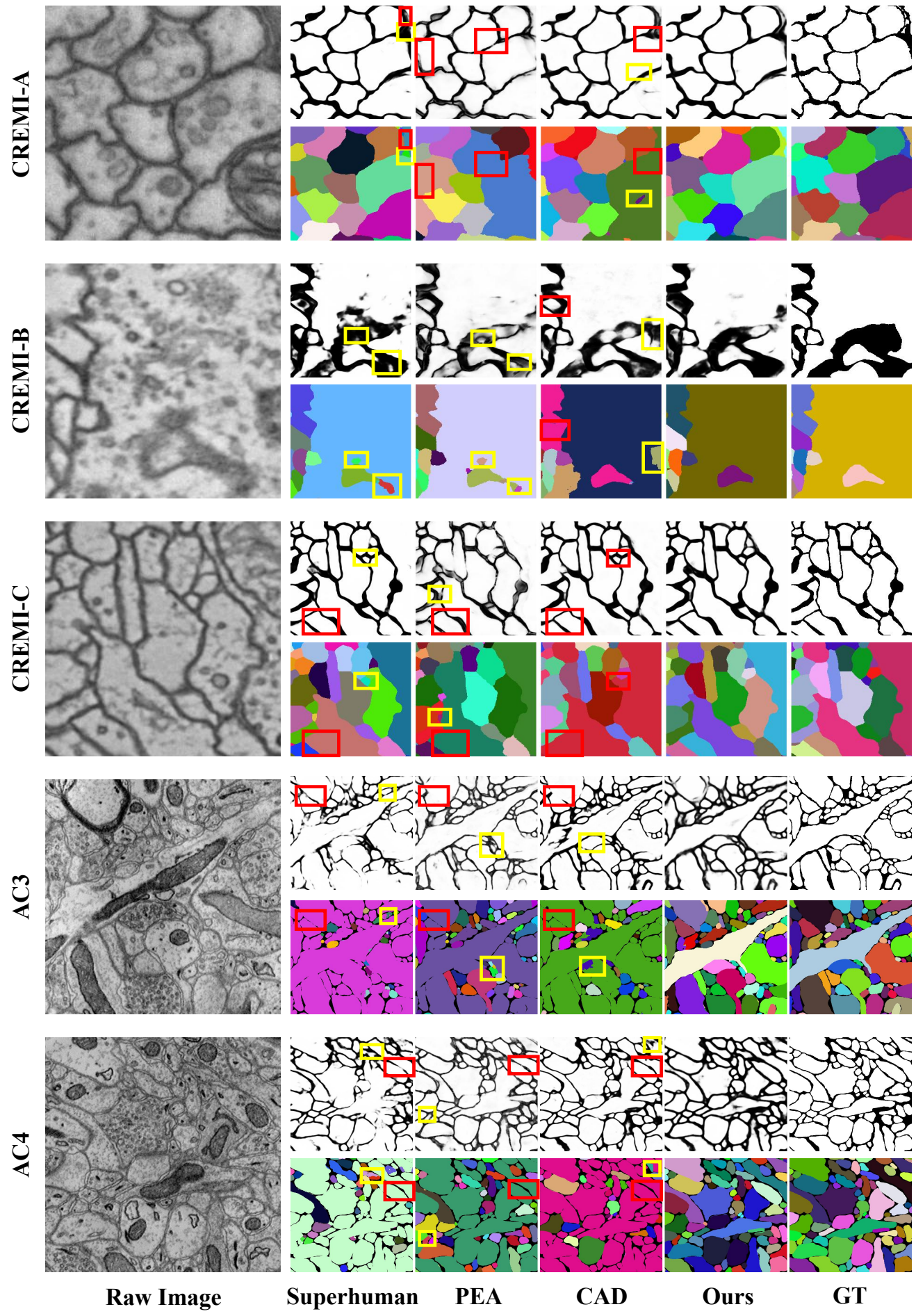

Figure S4 2D visual comparisons with supervised learning-based neuron segmentation methods on the CREMI and AC3/4 datasets. Red and yellow boxes highlight over-merging and over-splitting errors, respectively.

Additional Ablation Study

In this section, we present an additional ablation study to evaluate the key components of the copy-paste strategy as well as the impact of crop size scale.

Table S2. Ablation study of the bidirectional (BO) and similarity-based pair selection (SP) operations in our copy-paste method on the AC3 dataset with Waterz and LMC post-processing.

Method	BO	SP	Waterz		LMC	
			VOI↓	ARAND↓	VOI↓	ARAND↓
w/o BO	×	✓	1.418	0.209	1.343	0.127
w/o SP	✓	×	1.515	0.190	1.402	0.130
w/ BO+SP (**Ours**)	✓	✓	**1.048**	**0.099**	**1.218**	**0.098**

Ablation Study of Key Components in the Copy–Paste Strategy. To further validate the effectiveness of our copy-paste strategy, we perform an ablation study on the bidirectional operation (BO) and the similarity-based pair selection mechanism (SP) using the representative AC3 dataset. The results, summarized in Table S2 and Figure S5, demonstrate the individual and combined contributions of these two components. As shown in Table S2, removing either BO or SP clearly degrades performance, while enabling both operations (Ours) consistently achieves the best results under both post-processing pipelines, reaching VOI scores of 1.048 and 1.218 and ARAND scores of 0.099 and 0.098 with Waterz and LMC, respectively. Figure S5 further provides 2D qualitative comparisons of segmentation results. Compared with the variants without BO or SP, our copy-paste method (Ours) produces more accurate and coherent neuron segments, effectively reducing both over-splitting and over-merging errors. These results confirm the importance of incorporating both the bidirectional operation and the similarity-based pair selection mechanism.

Ablation Study of Crop Size Scale. To further investigate the impact of crop size scale on the performance of our copy-paste method, we conduct an ablation study on different crop size scale (β) on the representative AC3 dataset. The results are presented in Table S3 and Figure S6. In Table S3, as the crop size scale increases, the performance improves, with the best results achieved using a crop size scale of $2/3$ (Ours), where both VOI and ARAND are minimized. Specifically, with a crop size scale of $2/3$, our method achieves a VOI of 1.048 and ARAND of 0.099 under the Waterz post-processing pipeline, and a VOI of 1.218 and ARAND of 0.098 under LMC. In contrast, smaller crop size scale (e.g., $1/3$) result in less accurate segmentations, as reflected by higher VOI and ARAND values. Figure S6 further illustrates the 2D visual comparisons of segmentation results for different crop size scales on the AC3 dataset. These visual results corroborate the quantitative findings, where the $2/3$ crop size scale yields the most precise and coherent segmentation.

Table S3. Ablation study of the different crop size scale (β) in our copy-paste method on the representative AC3 dataset.

β (Scale)	Waterz		LMC	
	VOI↓	ARAND↓	VOI↓	ARAND↓
$1/3$	1.500	0.192	1.601	0.116
$1/2$	1.358	0.185	1.492	0.107
$2/3$	**1.048**	**0.099**	**1.218**	**0.098**
$5/6$	1.315	0.177	1.358	0.110

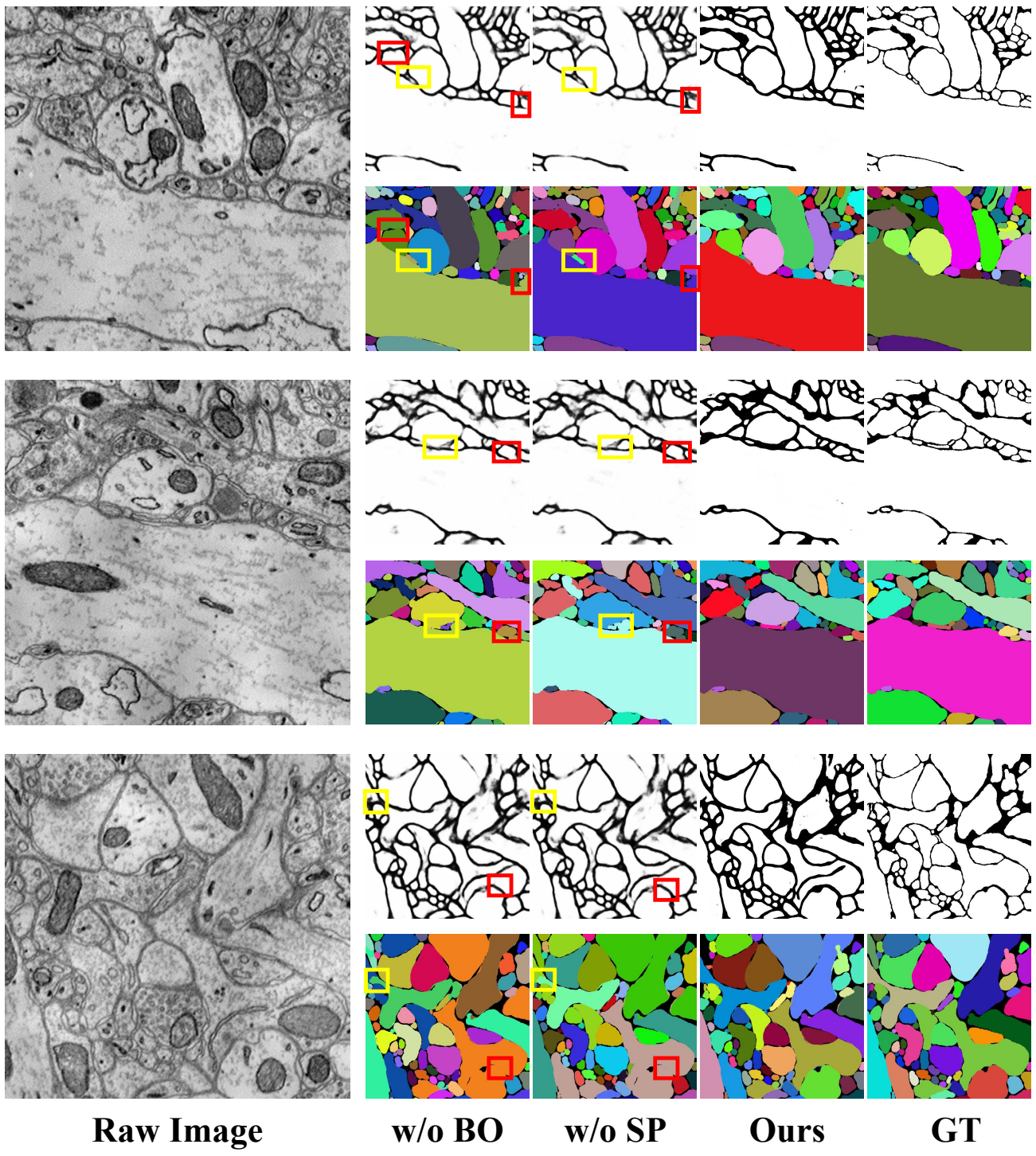

Figure S5 2D visual segmentation results for key components of the copy-paste strategy on the AC3 dataset. The left side shows the results without bidirectional (BO) and similarity-based pair selection (SP) operations, and the right side (Ours) shows the results with both operations enabled. Red and yellow boxes highlight over-merging and over-splitting errors, respectively.

Implementation Details.

We use the same 3D network architecture as in previous work to predict 3D affinity maps. Specifically, the 3D ResUNet model is employed for training, using the same 3D convolutional architecture adapted for volume data. The input volumes have dimensions of $18\times160\times160$ voxels^3, and are obtained by random cropping from the provided datasets. Data augmentation techniques such as intensity scaling, random rotations, elastic transformations, and noise augmentation are applied to the input data to improve model robustness. The training process uses the Adam optimizer, with a learning rate of $1e-4$, a batch size of 4, and a total of 300,000 iterations.During inference, the predicted affinity maps are converted into neuron segmentation masks using post-processing methods such as Waterz and LMC. *Additional implementation details are provided in the uploaded code included in the supplementary material.*

Detailed Description of Pseudocode

The overall procedure of our proposed method is summarized in Algorithm 1, which comprises three main stages: teacher network pre-training, student network training, and testing. In the teacher network pre-training stage, the teacher network Seg$(\cdot;\phi_t)$ is trained on perturbed unlabeled EM volumes to reconstruct the clean counterpart and learn meaningful structural representations, as defined in Eq. (4) and (5). During student network training stage, copy-paste volumes X_1 and X_2 are generated through similarity-based copy-paste augmentation (Eq. (14) and (12)). Pseudo-labels P_1 and P_2 are then produced by Seg$(\cdot;\phi_t)$ (Eq. (15)) and combined with ground-truth affinity maps to construct the final supervision signals S_1 and S_2 (Eq. (16)). The student network Seg$(\cdot;\phi_s)$ is optimized using the total loss $\mathcal{L}_{\text{total}}$ (Eq. (24)), while the teacher parameters θ_t are updated via exponential moving average (EMA) of the student weights to maintain stable pseudo-label generation. During the inference stage, the test volume T, cropped from V^T, is fed into the trained student network Seg$(\cdot;\phi_s)$ to predict the affinity map A. The predicted affinity map is subsequently post-processed to obtain the final neuron segmentation S.

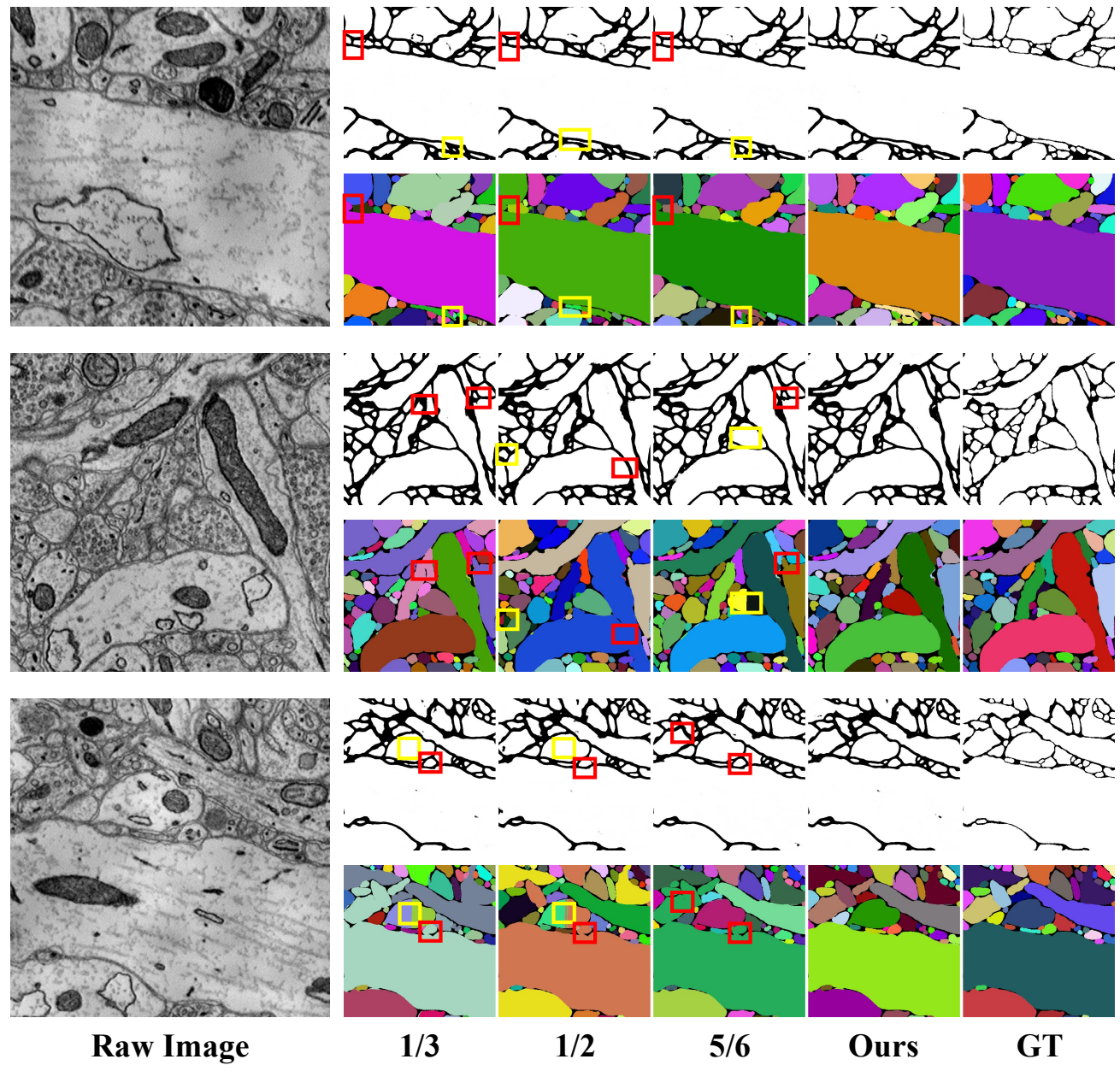

Figure S6 2D segmentation comparisons of the proposed SimCP-NS with different crop size scales on the AC3 dataset. From left to right: raw image, segmentation results with crop size scales $1/3$, $1/2$, and $5/6$, our method with crop size scale $2/3$, and ground truth. The yellow and red boxes highlight areas with over-splitting and over-merging errors, respectively.
